# Lipid Selectivity of Membrane Action of the Fragments of Fusion Peptides of Marburg and Ebola Viruses

**DOI:** 10.3390/ijms25189901

**Published:** 2024-09-13

**Authors:** Egor V. Shekunov, Svetlana S. Efimova, Lyudmila V. Kever, Tagir F. Ishmanov, Olga S. Ostroumova

**Affiliations:** Laboratory of Membrane and Ion Channel Modeling, Institute of Cytology of Russian Academy of Sciences, Tikhoretsky 4, 194064 Saint Petersburg, Russia; efimova@incras.ru (S.S.E.); mila.kever@mail.ru (L.V.K.); t_ishmanov@mail.ru (T.F.I.); ostroumova@incras.ru (O.S.O.)

**Keywords:** filoviruses, Ebola, Marburg, fusion peptide, lipid bilayer, membrane fusion, fusion inhibitors

## Abstract

The life cycle of Ebola and Marburg viruses includes a step of the virion envelope fusion with the cell membrane. Here, we analyzed whether the fusion of liposome membranes under the action of fragments of fusion peptides of Ebola and Marburg viruses depends on the composition of lipid vesicles. A fluorescence assay and electron microscopy were used to quantify the fusogenic activity of the virus fusion peptides and to identify the lipid determinants affecting membrane merging. Differential scanning calorimetry of lipid phase transitions revealed alterations in the physical properties of the lipid matrix produced by virus fusion peptides. Additionally, we found that plant polyphenols, quercetin, and myricetin inhibited vesicle fusion induced by the Marburg virus fusion peptide.

## 1. Introduction

Filoviruses constitute a family of enveloped viruses possessing a single-stranded, filamentous (-)RNA genome [1]. The first identified filovirus was the Marburg virus, discovered in 1967 [2]. Currently, the family includes two genera: Ebolavirus (EBOV) (encompassing Ebola, Sudan, Reston, Bundibugyo, and Tai Forest viruses) and Marburgvirus (MARV) [3]. Filoviruses are capable of infecting humans, non-human primates, and possibly other mammals, causing hemorrhagic fever in humans with a high mortality rate of up to 90% [1,4]. Due to their high lethality, filoviruses are classified as Category A priority pathogens under the NIAID research program [5]. Currently, there are no approved vaccines or treatments for filovirus infections [1].

The entry of the enveloped viruses into the host cell includes the fusion of viral and host cell lipid membranes using envelope glycoproteins [6]. These specialized proteins, known as fusion proteins, are amphipathic [7] and classified into three groups [8]. Filovirus fusion proteins belong to class I [1]. Class I fusion proteins feature a specific region, known as the fusion peptide, responsible for ensuring fusion between the virus and cell membranes [9]. Fusion peptides are predominantly hydrophobic sequences composed of 16–26 amino acids [10,11], enriched with alanine, glycine, and phenylalanine residues [12,13]. Despite differences in localization and amino acid sequence, class I viral fusion proteins exhibit a degree of homology and similarity in their mechanisms of action, suggesting a common evolutionary origin [14].

Filoviruses utilize the glycoprotein (GP), which is approximately 150 kDa in size and located on the virion surface, to mediate fusion with target cells [15]. This protein exists as a trimer, with each monomer comprising two subunits, GP1 and GP2, linked by a disulfide bond [16]. GP1 includes multiple regions, such as the receptor-binding domain (RBD) responsible for binding to cellular receptors, a glycan cap, and an O-linked highly glycosylated mucin-like domain [1]. RBDs of EBOV and MARV demonstrate 47% similarity, and their pseudovirions exhibit competitive infection of permissive cells [17]. GP2 contains an N-terminal fusion loop (FL), two heptad repeats (HR), a transmembrane domain, and a cytoplasmic tail [1]. The amino acid sequences of GP2 in EBOV and MARV show 60% identity [18]. The proposed fusion mechanism for filoviruses involves several steps. Initially, the viral particle binds to receptors on the cell surface (TIM-1, Axl) [19,20]. The preferred entry pathways for filoviruses include caveolin-mediated endocytosis, clathrin-mediated endocytosis, and micropinocytosis [1]. After cleavage of the mucin-like domain and glycan cap of GP1 by cathepsins in the acidic environment of the endosomes [21] and the explosion of RBD Niemann-Pick C1 (NPC1) cholesterol transporter [22], a highly hydrophobic FL sequence in GP2 is activated to promote primary fusion [1]. Once the FL inserts into the target membrane and destabilizes it [1], the two HR (NHR and CHR) of GP2 are exposed, forming a six-helix bundle, ultimately leading to the fusion of the viral and cellular membranes [23].

Lipid rafts are shown to play a significant role in EBOV entry [24,25]. Moreover, EBOV infection is accompanied by an increased production of ceramide, which is believed to promote it [26]. The high selectivity of the Ebola fusion peptide was also demonstrated in the work by Begoña Ruiz-Argüello [27]. In this study, the authors show that the fusogenic activity of the peptide depended on the presence of PI in the lipid mixture. Moreover, replacing this phospholipid with PG or PA abolished the activity. Triterpenoids were shown to inhibit the entry of EBOV and MARV via binding to the HR2 [28]. However, the sterol nature of tested triterpenoids might also assume the contribution of their membrane-associated effects to inhibition. These facts might be related to the lipid selectivity of membrane fusion induced by EBOV and MARV fusion peptides.

Since there are currently no approved vaccines or treatments for filovirus infections, the search for new antiviral drugs capable of infection prevention or suppressing the development of EBOV and MARV remains a relevant task [1]. In this regard, secondary plant metabolites are interesting candidates as potential antiviral agents. These molecules, due to their amphiphilic properties, are known to effectively intercalate into lipid membranes and thereby alter the biophysical properties of the bilayer [29,30]. Moreover, their effects strictly depend on the membrane lipid composition [31]. Previously, we demonstrated the ability of plant polyphenols and alkaloids to effectively inhibit membrane fusion induced by the fragments of fusion peptides of MERS-CoV and SARS-CoV-2 via modifying the elastic properties of the bilayers [32,33].

Here, we examined the possible lipid selectivity of the fusogenic action of FL of EBOV and MARV. For this purpose, several model lipid systems, based on liposomes of various composition and viral fusion peptide fragments, were developed, and fluorimetry, electron microscopy, and differential scanning microcalorimetry were applied. Additionally, we assessed the ability of several secondary plant metabolites to inhibit membrane fusion induced by the fragment of the MARV fusion peptide.

## 2. Results and Discussion

Using the simplified model systems based on liposomes of given lipid composition and short fragments of viral fusion peptides allows the identification of critical details of protein–lipid interactions and the structural–functional relationships between peptide activity and the amino acid sequence [34]. Applying this approach, the fusion of unilamellar lipid vesicles of different compositions mediated by 100 μM of _FP_MARV (_523_DLAAGLSWIPFFGPGIE_542_) and _FP_EBOV (_523_HNAAGIAWIPYFGPGAE_541_) was tested. As a positive control, the fragment of the gp41 fusion peptide of HIV was used (_512_AVGIGALFLGFLGAAGSTMGARS_534_) [23].

**Figure 1:**
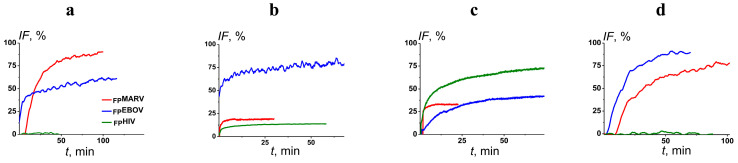
The time dependence of the relative calcein fluorescence leaked during fusion of liposomes composed of POPC/SM/CHOL (60/20/20 mol.%) (**a**), POPC/POPE/SM/CHOL (30/30/20/20 mol.%) (**b**), POPS/SM/CHOL (60/20/20 mol.%) (**c**), and POPC/CER/CHOL (60/20/20 mol.%) (**d**) induced by 100 μM of _FP_MARV, _FP_EBOV and _FP_HIV. The peptides were added at the initial moments. The relationship between color and fusion peptide type is shown in (**a**).

Considering that phosphatidylcholine (PC) is the most abundant lipid in the membranes of mammalian cells [35], the first lipid system for the evaluation of the ability of _FP_MARV, _FP_EBOV, and _FP_HIV to induce membrane fusion was POPC/SM/CHOL (60/20/20 mol.%). Figure 1a demonstrates the time courses of calcein fluorescence due to liposome fusion induced by various fusion peptides. _FP_MARV and _FP_EBOV induced the fusion of POPC/SM/CHOL liposomes, reaching about 85 and 60%, respectively, while _FP_HIV did not practically exhibit significant fusogenic activity under these conditions. Table 1 presents the mean maximum values of fluorescence intensity of released calcein mediated by 100 μM of different fusion peptides (*IF_max_*).

**Figure 2:**
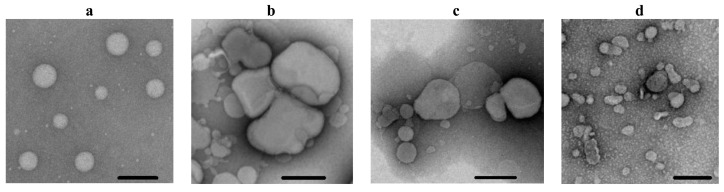
Electron microphotographs of POPC/SM/CHOL (60/20/20 mol.%) liposomes before (**a**) and after incubation with 100 μM of _FP_MARV (**b**), _FP_EBOV (**c**), and _FP_HIV (**d**). Black scale bars represent 200 nm.

Additionally, electron microscopy was used to visualize the different abilities of _FP_MARV, _FP_EBOV, and _FP_HIV to induce the fusion and aggregation of POPC/SM/CHOL liposomes. Figure 2 shows the typical micrographs of POPC/SM/CHOL liposomes before (a) and after incubation with 100 μM of _FP_MARV (b), _FP_EBOV (c), and _FP_HIV (d). In the absence of any fusion peptides, the diameter of unmodified POPC/SM/CHOL liposomes was estimated to be 125 ± 30 nm. The addition of _FP_MARV and _FP_EBOV led to the deformation and aggregation of liposomes and an increase in the size of observed lipid structures up to 240 ± 132 and 211 ± 83 nm, respectively (Figure 2b,c). _FP_HIV produced lower vesicle aggregation and caused a slight enlargement in the size of lipid structures (171 ± 79 nm) (Figure 2d).

According to the literature data, the monolayer spontaneous curvature can significantly affect membrane fusion [36], so we introduced into the membrane the cone-shaped lipid phosphatidylethanolamine (PE), which is believed to promote fusogenic activity by reducing the energy cost of the formation of fusion intermediates characterized by high negative curvature [37,38]. It should be noted, that, along with PC, PE is also widespread in the outer layer of mammalian plasma membranes [39]. As expected, _FP_HIV produced a more significant fusion of POPC/POPE/SM/CHOL (30/30/20/20 mol.%) liposomes compared to POPC/SM/CHOL (60/20/20 mol.%) vesicles (by about 3 times) (Figure 1b, Table 1). _FP_EBOV fusion activity was slightly potentiated by the addition of POPE (by about 1.2 times) than _FP_HIV (Figure 1b, Table 1). Surprisingly, the replacement of the portion of cylindrical POPC for POPE led to a 5-fold decrease in the fusogenic action of _FP_MARV (Figure 1b, Table 1). This clearly indicated the dramatic difference in the mechanisms underlying the fusion induced by _FP_MARV and _FP_HIV. Viral fusion peptides used several mechanisms to induce membrane fusion, including the dehydration of lipid head groups, alteration in acyl chain order, and changes in the membrane spontaneous curvature [34,40,41]. Considering the reduction in the activity of _FP_MARV during POPE addition into membrane composition and the small potentiating effect of POPE on _FP_EBOV-mediated merging (Table 1), we proposed that spontaneous curvature stress was not a key factor determining the fusion under the action of the peptides. It is also known that membranes composed of PE are more weakly hydrated than PC [42,43]. Therefore, we hypothesized that _FP_MARV induced membrane fusion via the dehydration of lipid bilayers. Removing water molecules between membranes makes it easier to achieve the critical (minimum) distance, which is necessary to initiate merging and stalk formation due to the diminishing hydration repulsion [44,45]. For example, dehydration also plays a key role in the SNARE-induced fusion [46]. Under conditions of high hydration, highly hydrophobic peptides (including _FP_MARV and _FP_EBOV) should self-associate on the membrane surface to reduce the number of contacts with water molecules. The self-association of the fusion peptides is known to highly impact the fusogenic activity of several fusion peptides, and oligomers showed a higher ability to induce fusion compared with monomers [47,48,49]. The difference in POPE effects on _FP_MARV and _FP_EBOV-induced fusion might be related to the difference in the type and localization of charged and polar amino acid residues in the peptides determining their conformations.

Electrostatic interactions between the fusion peptides and negatively charged membrane lipids also play an important role in membrane fusion [50]. To investigate this opportunity to regulate the fusogenicity of _FP_MARV and _FP_EBOV, anionic lipid phosphatidylserine (PS) was included in the lipid composition of the vesicles. The endosomal membranes are enriched with PS [51], and it is important for the entry of certain viruses, in particular, HIV [52,53]. Figure 1 shows the effects of _FP_MARV, _FP_EBOV, and _FP_HIV on the fusion of liposomes composed of POPS/SM/CHOL (60/20/20 mol.%). The replacement of neutral POPC for negatively charged POPS led to a 1.5–1.8-times reduction in the fusogenic activity of _FP_MARV and _FP_EBOV, while it caused a sharp increase in _FP_HIV action (Table 1). A decrease in the activity of _FP_MARV might be related to electrostatic repulsion between the negatively charged bilayer and the fusion peptide that contains negatively charged residues of aspartic and glutamic acids. At the same time, _FP_HIV showed a high selectivity toward the anionic lipids due to the presence of cationic arginine in its structure. Our results are in agreement with previous findings by Zaitseva et al., which indicated the key role of the exposing of PS on cell membrane surface in HIV entry [53]. These results are also consistent with earlier studies, which indicated that the presence of anionic lipids in the membrane allows _FP_HIV to adopt an appropriate conformation associated with increased fusogenicity [54,55,56].

It is well known that lipid phase segregation is an important factor in the fusion of enveloped viruses [57], including filoviruses [24] and HIV [42]. For example, the EBOV infection activates acid sphingomyelinase, increases the production of ceramides, and leads to the formation of membrane domains with a high ceramide content, which contribute to further infection [26]. Ceramide-enriched, membrane-ordered domains also enhance the fusion activity of SARS-CoV-2 [58]. To simulate a situation, SM in the membrane composition was replaced by ceramide (CER). _FP_MARV and _FP_HIV did not exhibit noticeable selectivity between POPC/SM/CHOL and POPC/CER/CHOL (60/20/20 mol.%) systems. However, the inclusion of CER instead of SM produced a slight potentiating effect on _FP_EBOV activity (Figure 1 and Table 1).

**Figure 3:**
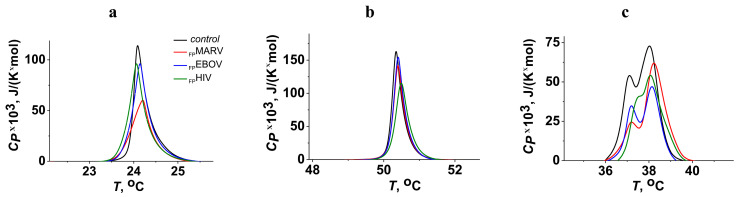
Heating thermograms of DMPC (**a**), DMPE (**b**), and DMPS (**c**) in the absence (*black curves*) and presence of _FP_MARV (*red curves*), _FP_EBOV (*blue curves*), and _FP_HIV (*green curves*) at lipid:peptide molar ratio of 25:1. The relationship between the color and peptide type is shown in (**a**).

Membrane elastic properties and lipid packing play a huge role in membrane-fusion processes [34,59]. For this reason, we studied the phase behavior of different lipids in the presence of _FP_MARV, _FP_EBOV, and _FP_HIV. We varied the type of nitrogenous base in the lipid head (DMPC, DMPE, and DMPS) and the length of hydrocarbon chains (DTPC, DMPC, and DPPC). The thermotropic behavior of lipids was characterized by the temperature of melting (*T_m_*), the width of the main phase transition peak (Δ*T_b_*), and the enthalpy of the main phase transition (Δ*H*). Figure 3 presents the examples of heating thermograms of DMPC, DMPE, and DMPS in the absence (control) and presence of tested fusion peptides at a lipid:peptide molar ratio of 25:1. Table 2 presents the mean parameters of DMPC, DMPE, and DMPS thermograms in the presence of _FP_MARV, _FP_EBOV, and _FP_HIV. The addition of _FP_EBOV and _FP_HIV did not practically affect the main phase transition of DMPC (*T_m_*, Δ*T_b_*, and Δ*H*), while _FP_MARV slightly increased Δ*T_b_* and decreased Δ*H* (Figure 3a, Table 2). This was in agreement with the highest ability of _FP_MARV to affect POPC/SM/CHOL liposomes among the tested peptides (Figure 1a and Table 1). The replacement of DMPC with DMPE led to a decrease and increase in _FP_MARV and _FP_HIV effects, respectively (Figure 3b, Table 2). So, in contrast to DMPC, _FP_HIV was able to significantly increase the melting temperature of DMPE and produced a dramatic decrease in transition enthalpy. Interestingly, the inclusion of POPE also decreased and increased the fusogenic activity of _FP_MARV and _FP_HIV, respectively (Figure 1b, Table 1).

All tested fusion peptides were able to influence DMPS melting (Figure 3c and Table 2). Figure 3c demonstrates that the main phase transition of pure DMPS had a biphasic nature with low (*T_m__*_1_) and high (*T_m__*_2_) melting components. It is known that this is associated with the different protonation of serine and various degrees of lipid hydration in these states, and the lower the hydration, the higher the melting point [60,61]. The addition of _FP_MARV and _FP_EBOV led to an increase in the melting point (*T_m__*_2_) and relative impact (Δ*H*_2_/Δ*H*_1_) of high melting components (Figure 3c and Table 2).This indicates that the peptides caused the dehydration of PS membranes. This finding was in good agreement with the above assumption of the dehydrating mode of fusogenic action of _FP_MARV and _FP_EBOV. _FP_HIV demonstrated a qualitatively different effect: it reduced deconvolution by joining two components in one peak (Figure 3c and Table 2).

**Figure 4:**
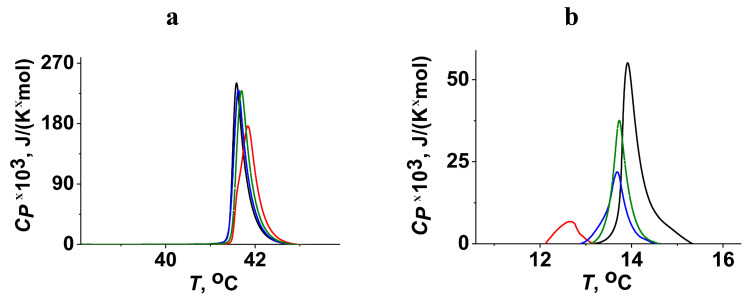
Heating thermograms of DPPC (**a**) and DTPC (**b**) in the absence (*black curves*) and presence of _FP_MARV (*red curves*), _FP_EBOV (*blue curves*), and _FP_HIV (*green curves*) at lipid:peptide molar ratio of 25:1. The relationship between the color and peptide type is shown in Figure 3a.

We also examined the dependence of the effects of the fusion peptides on PC melting on the length of acyl chains. Figure 4 presents the typical heating thermograms of DPPC (a) and DTPC (b) in the absence and in the presence of _FP_MARV, _FP_EBOV, and _FP_HIV at a lipid:peptide molar ratio of 25:1. The increase in the chain length due to replacement of DMPC (14:0) to DPPC (16:0) had virtually no effect on the interaction of peptides with the membrane (Figure 3a and Figure 4a). Moreover, the _FP_MARV-induced decrease in Δ*H* of DPPC melting was even lower than that of DMPC. The diminishing tail length from 14 (DMPC) to 13 acyl units (DTPC) had dramatic consequences for peptide action: a noticeable shift of the transition point (*T_m_*) to the lower temperatures and a significant decrease in peak area (i.e., Δ*H*) were observed (Figure 3a and Figure 4b). The ability to decrease *T_m_* and Δ*H* decreased in the order of _FP_MARV, _FP_EBOV, and _FP_HIV (Figure 4b). The pronounced decrease in *T_m_* and Δ*H* of the DTPC transition produced by _FP_MARV might indicate that the fusion peptide promotes lipid tail protrusions (i.e., the movement of the lipid acyl chain toward the phosphate group due to setting more hydrophobic conditions on the membrane surface at dehydration). The occurrence of such protrusions was demonstrated in several studies with fragments of different fusion peptides, including influenza [62] and parainfluenza virus peptides [48]. The lower probability of the formation of such defects in the presence of the peptide on the surface of DMPC and DPPC bilayers may be due to their greater initial hydration [63]. In good agreement with calorimetry data, an increase in the lipid chain length (by replacing POPC (16:0–18:1) for 1,2-dieicosenoyl-*sn*-glycero-3-phosphocholine (20:1 (Cis) PC) in liposome composition) inhibited the activity of _FP_MARV in a fusion fluorescence assay (the maximum *IF*-value was equal to 18%).

**Figure 5:**
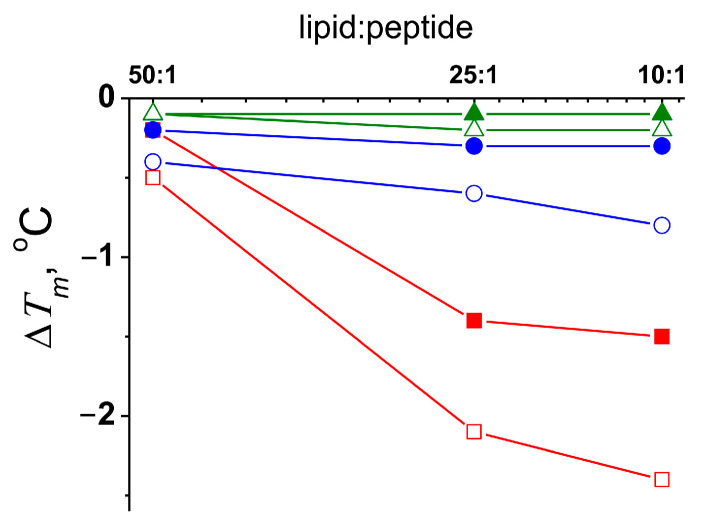
The changes in the melting temperature (∆*T_m_*) of DTPC in the presence of _FP_MARV (*red curves*), _FP_EBOV (*blue curves*), and _FP_HIV (*green curves*) at lipid:peptide molar ratio of 50:1, 25:1, and 10:1. Closed and open symbols correspond to heating and cooling thermogram, respectively.

An analysis of the heating and cooling thermograms of DTPC in the absence and presence of the tested fusion peptides was carried out (Figure 5). It was found that _FP_MARV and _FP_EBOV more significantly affected the freezing temperature than the melting point. This indicated the preferential interaction of _FP_MARV and _FP_EBOV with the liquid–crystalline lipid phase than with the gel state. _FP_HIV produced close effects with the transition temperature during cooling and heating (Figure 5). These data were consistent with the results found by Yang et al. [64], which suggested that HIV fusion action arises at the boundaries of ordered/disordered membrane domains.

The elucidation of the mechanisms of the fusogenic action of the MARV fusion peptide allows us to identify possible approaches to its inhibition. Given the established key role of dehydration in _FP_MARV-induced fusion, we have hypothesized that small hydrophilic molecules, such as plant flavonols, might inhibit this process.

In contrast, compounds that act primarily on the elastic properties of the membrane and thus inhibit fusion by other viral peptides, in particular the plant alkaloid piperine [33], should not have a similar effect. To analyze the inhibitory activity of secondary plant metabolites, we applied calcein leakage.

The number of hydroxyl groups decreases in the series of tested flavonols: myricetin (6), quercetin (5), and fisetin (4), which is expressed in an appropriate increase in the partitioning coefficient between octanol and water (*LogD*) (Table 3).

**Figure 6:**
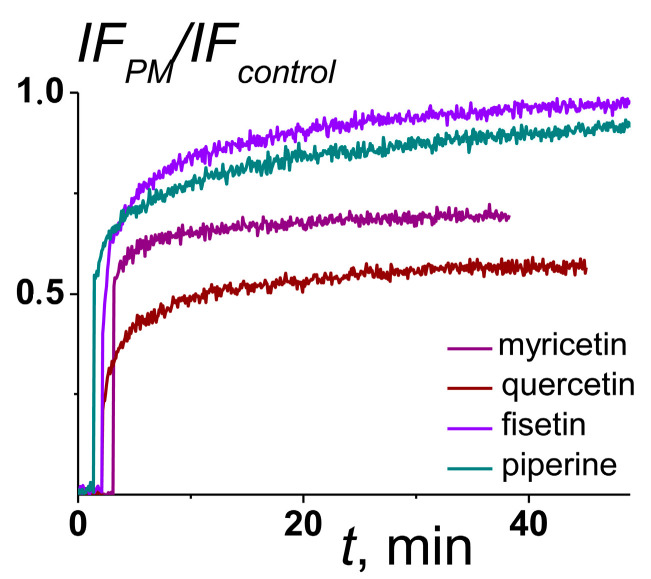
The time dependence of the ratio between relative calcein fluorescence leaked at _FP_MARV-induced fusion of POPC/SM/CHOL (60/20/20 mol.%) liposomes in the presence of certain plant metabolite and maximum relative calcein leakage due to peptide-mediated vesicle fusion in the absence of any membrane-active compounds. The peptide was added at the initial moments up to 100 μM. Liposomes have been incubated with 20 μM of quercetin, myricetin, or fisetin and 400 μM of piperine for 30 min before the addition of fusion peptide. The relationship between color and plant metabolite is shown in the figure.

Figure 6 presents the effects of plant metabolites on the kinetics of calcein release due to the fusion of POPC/SM/CHOL (60/20/20 mol.%) liposomes mediated by 100 μM of _FP_MARV. Fisetin and piperine did not demonstrate inhibitory activity (*IA*), while myricetin and quercetin were able to prevent the vesicle fusion induced by _FP_MARV. Table 3 shows the mean *IA* values. Myricetin and quercetin inhibited the _FP_MARV-induced liposome fusion by 30–40% (Table 3). More probable, myricetin and quercetin counteracted the dehydrating action of _FP_MARV due to the high hydrophilicity of their molecules, which led to the inhibition of the peptide-promoted fusion of lipid vesicles. Fisetin did not have a similar effect, which is quite expected given that its molecule is less hydroxylated (Table 3). It should be noted that quercetin and myricetin were also shown to inhibit calcium-induced membrane fusion [32]. Piperine did not suppress _FP_MARV-associated membrane fusion (*IA* was less than 7%) (Table 3). The alkaloid is characterized by high hydrophobicity (Table 3), which allows it to penetrate into the bilayer hydrocarbon core and pronouncedly act on elastic stress, while it should weakly affect polar interactions on the membrane surface. Thus, the ability of secondary plant metabolites to inhibit the fusogenic effect of _FP_MARV correlated well with the hydrophilicity of their molecules.

The literature data indicated the promising antiviral activity of myricetin and quercetin. For example, myricetin was shown to exhibit inhibiting activity against the infectious bronchitis virus and human immunodeficiency virus 1 by affecting viral papain-like protease and reverse transcriptase, respectively [65,66]. Similarly, quercetin was shown to suppress hepatitis C virus and Ebola infections by inactivating the viral NS3 protease and multifunctional protein VP24 binding to the type-I interferon system, respectively [67,68]. Quercetin also demonstrated significant inhibitory activity against Dengue virus 2, although the exact mechanism remained unknown [69]. Quercetin inhibited herpes simplex virus 1 entry by blocking virion binding to host cells [70]. Quercetin 3-β-O-d-glucoside was also shown to be capable of suppressing the Ebola and Sudan viruses at early steps of viral entry, specifically fusion with the cell [71]. These results indicated that plant flavonols can inhibit virus infection through multiple mechanisms, and a detailed analysis of the impact of inhibiting the fusion of viral and cell host membranes due to altering the membrane hydration by the compounds should be estimated. Our findings indicate that highly hydroxylated flavonoids may be considered as possible components of combination therapy to prevent MARV infection. The use of fusion inhibitors with a lipid-associated mechanism of action is a promising direction for treating viral infections [72]. There is evidence that the molecule LJ001 demonstrates high antiviral activity against a large number of enveloped viruses, including the Ebola virus [73]. This compound shows high selectivity for viral lipid membranes and is non-toxic in vitro and in vivo [73]. According to Agarwal et al., steroidal amines inhibit activity against the influenza virus due to their molecules modulating lipid rafts [74]. The sphingosine-like compound J582C promotes a decrease in the membrane order, which leads to a reduction in the density of the HIV lipid envelope, thus preventing the virus from fusing with the cell [75].

## 3. Materials and Methods

### 3.1. Materials

All reagents used were of high purity.

#### 3.1.1. Membrane-Forming Lipids 

1-palmitoyl-2-oleoyl-*sn*-glycero-3-phosphocholine (POPC), 1,2-dieicosenoyl-*sn*-glycero-3-phosphocholine, 1,2-ditridecanoyl-*sn*-glycero-3-phosphocholine (DTPC), 1,2-dimyristoyl-*sn*-glycero-3-phosphocholine (DMPC), 1,2-dipalmitoyl-*sn*-glycero-3-phosphocholine (DPPC), 1-palmitoyl-2-oleoyl-*sn*-glycero-3-phosphoethanolamine (POPE), 1,2-dimyristoyl-sn-glycero-3-phosphoethanolamine (DMPE), 1-palmitoyl-2-oleoyl-*sn*-glycero-3-phospho-L-serine (POPS), 1,2-dimyristoyl-*sn*-glycero-3-phospho-L-serine (DMPS), sphingomyelin (Brain, porcine) (SM), ceramide (Brain, porcine) (CER), and cholesterol (CHOL) were purchased from Avanti Polar Lipids Avanti Polar Lipids, Inc., Alabaster, AL, USA) and solubilized in analytical-grade chloroform.

#### 3.1.2. Fusion Peptides

The peptides were synthesized via solid-phase synthesis (purity ≥98%) by Elabscience Biotechnology Inc. This study utilized the constructs _FP_MARV (_523_DLAAGLSWIPFFGPGIE_542_) and _FP_EBOV (_523_HNAAGIAWIPYFGPGAE_541_). The amino acid sequences of the peptides are homologous to the FL of EBOV (strain Sudan/Uganda-00) (NCBI:txid386033) and MARV (strain Lake Victoria/Angola-05) (NCBI:txid378830), respectively [76]. We also used the fragment of gp41 peptide from the human immunodeficiency virus as a well-studied fusion peptide (_FP_HIV, _512_AVGIGALFLGFLGAAGSTMGARS_534_) (NCBI:txid11676) [23]. The amino acid sequences of the samples were confirmed by MALDI-TOF mass spectrometry. Lyophilized samples were dissolved in dimethyl sulfoxide (DMSO) at room temperature.

#### 3.1.3. Secondary Plant Metabolites

Piperine, quercetin, myricetin, and fisetin were purchased from Sigma-Aldrich Company Ltd. (Merck KGaA, Darmstadt, Germany).

#### 3.1.4. Other Reagents

Calcein, NaCl, NaOH, HCl, HEPES, EDTA, phosphate-buffered saline (PBS), Triton X-100, chloroform, DMSO, and sephadex G-50 were purchased from Sigma-Aldrich Company Ltd. (Merck KGaA, Darmstadt, Germany).

### 3.2. Methods

#### 3.2.1. MembraneFusion Assay

##### Lipid Vesicle Preparation

Unilamellar vesicles (UVs) composed of POPC/SM/CHOL (60/20/20 mol.%), POPC/POPE/SM/CHOL (30/30/20/20 mol.%), POPS/SM/CHOL (60/20/20 mol.%), or POPC/CER/CHOL (60/20/20 mol.%) were used as the model membrane systems. The appropriate lipid mixtures at a concentration of 3 mM were dissolved in chloroform in glass tubes, followed by solvent removealunder nitrogen for about 5 min. The resulting lipid film was rehydrated in 300μLof buffer containing 35 mM of calcein, 10 mM of HEPES, and pH of 7.4. Five freeze–thaw cycles were then performed. The resulting vesicle suspension was extruded through polycarbonate filters of Ø100 nm thirteen times. Unencapsulated calcein was removed via gel filtration using sephadex G-50. The replacement buffer consisted of 150 mM of NaCl, 10 mM of HEPES, 1 mM of EDTA, and pH of 7.4.

##### Calcein Leakage Assay

Fusion/aggregation of vesicles induced by various fusion peptides was assessed via calcein leakage. Calcein, encapsulated within vesicles at millimolar concentrations, exhibits low fluorescence due to self-quenching. A change in membrane permeability for a fluorescent dye during topological rearrangements of membranes under the influence of fusion peptides [77] leads to the release of calcein into the surrounding solution, where it fluoresces. Thus, the addition of 100 μM of _FP_MARV, _FP_EBOV, or _FP_HIV leads to an increase in fluorescence intensity, which can be measured to determine the extent of vesicle fusion/aggregation. Calcein fluorescence (excitation at 490 nm; emission at 520 nm) was monitored using a Fluorat-02-Panorama at 25 °C. At the end of the measurements, Triton X-100 was added to each sample to a final concentration of 1% to fully disengage the marker and to measure the maximum fluorescence.

To describe vesicle fusion/aggregation, the obtained data were processed using an equation based on the percentage increase in calcein fluorescence over time (*IF*, %):

(1) $$IF\;=\;\frac{{\mathrm{RF}}_{t}-{\mathrm{RF}}_{0}}{{\mathrm{RF}}_{\infty }/0.9-{\mathrm{RF}}_{0}}\cdot 100\%$$

where *RF*_0_, *RF_t_*, and*RF_∞_* are the fluorescence intensity of calcein at different times: 0, *t,* and ∞, respectively. *RF*_∞_ was taken to be the value after adding Triton X-100, which caused complete release of calcein. To account for sample dilution, a factor of 0.9 was introduced.

*IF*-values were averaged from 2–5 independent experiments for each tested system (mean value ± standard error) (*p* ≤ 0.05).

##### Analysis of Antifusogenic Activity of Secondary Plant Metabolites

To analyze the inhibitory activity of secondary plant metabolites, we used the calcein leakage method described above. Liposomes composed of POPC/SM/CHOL (60/20/20 mol.%) were pre-incubated for 30 min with 20 μM of quercetin, myricetin, and fisetin or 400 μM of piperine. Following this, _FP_MARV was added at a concentration of 100 μM.

The inhibitory activity of the secondary plant metabolites (*IA*, %) was described using the following equation:

(2) $$IA\;=\;\frac{{\mathrm{IF}}_{0}-{\mathrm{IF}}_{\mathrm{PM}}\;}{{\mathrm{IF}}_{0}}\cdot 100\%$$

where *IF*_0_ and *IF_PM_* represent the maximum relative fluorescence intensity induced by the addition of _FP_MARV in the absence and in the presence of the tested secondary plant metabolite, respectively.

*IA*-values were averaged from 2–5 independent experiments (mean value ± standard error) (*p* ≤ 0.05).

#### 3.2.2. Electron Microscopy (EM)

##### Lipid Vesicle Preparation

Lipid mixture (POPC/SM/CHOL (60/20/20 mol.%)) was suspended in a mixture of chloroform (67 vol.%) and methanol (33 vol.%). The lipid concentration was equal to 250 µM. The resulting solution was evaporated in a vacuum rotary evaporator at room temperature for 60 min. Further, lipid film was dispersed in a buffer (0.15 M of NaCl, 10 mM of HEPES, and pH of 7.4) and was exposed to ultrasound for about 3 min.

##### EM Experiments

To obtain electron micrographs, the method of negative staining with a 1% aqueous solution of uranyl acetate within 20 s was used. The vesicles in the absence (control) and presence of 100 μM of _FP_MARV, _FP_EBOV, and _FP_HIV (pre-incubated for 30 min) were placed on copper grids coated with a collodion film substrate. Electron micrographs of the liposomes were obtained using a transmission electron microscope Libra 120 (Carl Zeiss, Jena, Germany).

The size of lipid structures was averaged from 2 independent experiments for each tested system (mean value ± standard deviation) (*p* ≤ 0.05).

#### 3.2.3. Differential Scanning Calorimetry (DSC)

##### Lipid Vesicle Preparation

Unilamellar vesicles composed of DTPC, DMPC, DPPC, DMPS, or DMPE were prepared using electroformation method using the Nanion vesicle *prep pro* for 1 h at alternating voltage (3 V, 10 Hz) and 25 °C (DTPC), 35 °C (DMPC), 41 °C (DPPC), 55 °C (DMPS), or 55 °C (DMPE). The resulting liposome suspension contained 5mM of lipid and was buffered by 5 mM of HEPES, with pH of 7.4.

##### DSC Experiments

The control samples were not modified. _FP_MARV, _FP_EBOV, or _FP_HIV were added to aliquots to obtain the lipid:peptide molar ratio of 50:1, 25:1, and 10:1. DSC experiments were performed by a μDSC 7EVO microcalorimeter (Setaram, France) with heating and cooling rates of 0.2 and 0.3 °C/min, respectively. The samples with peptides were stabilized for about 30 min at room temperature. The reversibility of the thermal transitions was assessed by reheating the sample immediately after the cooling step from the previous scan. Thermogram analysis was performed using the Calisto software package. The peaks on the thermograms were characterized by the temperature of the lipid melting (*T_m_*), the peak width corresponding to the temperature difference between the upper (beginning) and lower (end) boundaries of the main phase transition (∆*T_b_*), and the enthalpy of the transition (Δ*H*). In the case of DMPS, two components of main transition characterized by lower (*T_m__*_1_) and higher (*T_m__*_2_) melting temperature were analyzed separately. The ratio between the enthalpy of low- and high-melting components (Δ*H*_2_/Δ*H*_1_) was also determined.

The Δ*T_m_*, ΔΔ*T_b_*, ΔΔ*H*, andδ(Δ*H*_2_/Δ*H*_1_) values were averaged over 2–3 independent experiments and presented as mean value ± standard deviation (*p* ≤ 0.05).

## Figures and Tables

**Table 1:** The dependence of fusogenic ability of _FP_MARV, _FP_EBOV, and _FP_HIV (*IF_max_*, %) on lipid composition of unilamellar lipid vesicles.

FP Fragment *	Lipid Composition			
	POPC/SM/CHOL (60/20/20 mol.%)	POPC/POPE/SM/CHOL (30/30/20/20 mol.%)	POPS/SM/CHOL (60/20/20 mol.%)	POPC/CER/CHOL (60/20/20 mol.%)
_FP_MARV	74 ± 13	18 ± 6	50 ± 14	71 ± 9
_FP_EBOV	65 ± 16	81 ± 5	37 ± 10	86 ± 10
_FP_HIV	5 ± 4	15 ± 4	87 ± 3	2 ± 1

* _FP_MARV (_523_DLAAGLSWIPFFGPGIE_542_), _FP_EBOV (_523_HNAAGIAWIPYFGPGAE_541_), and _FP_HIV (_512_AVGIGALFLGFLGAAGSTMGARS_534_) at 100 μM.

**Table 2:** The effects of _FP_MARV, _FP_EBOV, and _FP_HIV on the thermotropic behavior of different lipids.

FP Fragment	DMPC			DMPE			DMPS		
	Δ*T_m_*, °C	Δ∆*T_b_*, °C	ΔΔ*H*, kcal/mol	Δ*T_m_*, °C	Δ∆*T_b_*, °C	ΔΔ*H*, kcal/mol	Δ*T_m__*_1_, °C	Δ*T_m__*_2_, °C	δ(Δ*H*_2_/Δ*H*_1_)
_FP_MARV	0.1 ± 0.1	0.4 ± 0.1	−3.0 ± 0.3	0.3 ± 0.1	0.1 ± 0.1	−0.5 ± 0.2	0.1 ± 0.1	0.3 ± 0.1	1.6 ± 0.1
_FP_EBOV	0	0.1 ± 0.1	−0.2 ± 0.1	0.2 ± 0.1	0	−0.6 ± 0.2	0.1 ± 0.1	0.1 ± 0.1	1.2 ± 0.2
_FP_HIV	0	0.1 ± 0.1	−0.2 ± 0.1	0.4 ± 0.2	0	−16.2 ± 3.5	0.4 ± 0.1	0	1.0 ± 0.1

The lipid:peptide molar ratio was equal to 25:1; Δ*T_m_*, ΔΔ*T_b_*—the peptide-induced changes in the maximum temperature of the *L_β_*/*L_α_* transition of DMPC, DMPE, and DMPS and the temperature difference between the upper (onset) and lower (completion) boundary of the main phase transition of the lipids. The *T_m_* of unmodified DMPC and DMPE was equal to 24.1 ± 0.2 °C and 50.2 ± 0.1 °C, respectively. The *T_m_*__1_ and *T_m_*__2_ of DMPS were equal to 36.9 ± 0.1 °C and 37.6 ± 0.2 °C, respectively. Δ*T_b_* of unmodified DMPC and DMPE was equal to 2.3 ± 0.5 and 2.2 ± 0.3 °C, respectively; ΔΔ*H*—the changes in the enthalpy of main transition of DMPC and DMPE in the presence of the peptides. The Δ*H* of unmodified DMPC and DMPE was equal to 15.8 ± 1.1 and 37.2 ± 1.4 kcal/mol, respectively; δ(Δ*H*_2_/Δ*H*_1_) is peptide-induced relative alteration in the ratio of enthalpies of low- and high-melting components of DMPS main transition compared to control.

**Table 3:** The inhibitory activity (*IA*, %) of different plant metabolites on the fusogenic activity of _FP_MARV in POPC/SM/CHOL system.

Plant Metabolite	Chemical Structure	*LogD* ^#^	*IA*, %
myricetin	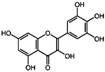	1.06	30 ± 2
quercetin	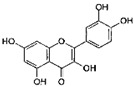	1.46	38 ± 4
fisetin	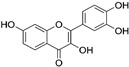	2.3	2 ± 2
piperine	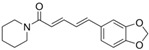	2.78	3 ± 1

^#^ The values of the logarithm of octanol/water distribution coefficient at pH 7.4 (*LogD*) were predicted with ChemAxon (https://chemaxon.com).

## Data Availability

Data are contained within the article.
